# Welding, Joining, and Coating of Metallic Materials

**DOI:** 10.3390/ma13112640

**Published:** 2020-06-10

**Authors:** Michael Zinigrad, Konstantin Borodianskiy

**Affiliations:** Department of Chemical Engineering, Ariel University, Ariel 40700, Israel; zinigrad@ariel.ac.il

**Keywords:** welding, solidification, coating, metals, materials properties, build-up

## Abstract

Welding, joining, and coating of metallic materials are among the most applicable fabrication processes in modern metallurgy. Welding or joining is the manufacture of a metal one-body workpiece from several pieces. Coating is the process of production of metallic substrate with required properties of the surface. A long list of specific techniques is studied during schooling and applied in industry; several include resistant spot, laser or friction welding, micro arc oxidation (MAO), chemical vapor deposition (CVD), and physical vapor deposition (PVD), among others. This Special Issue presents 21 recent developments in the field of welding, joining, and coating of various metallic materials namely, Ti and Mg alloys, different types of steel, intermetallics, and shape memory alloys.

Metals and alloys fabrication, known as metallurgy, has been known since ancient times as the art of making tools and devices for practical applications. This is a scientific field of research that focuses on the study of metals’ properties and production from the Stone Age through the Bronze Age and the Iron Age to the modern age of today. Modern metallurgy focuses not only on products for daily use but also on the fabrication of novel materials for aerospace, automotive, marine, nuclear, electric, electronic, and other industries. Investigation of the metallic structure is of the highest scientific interest since it has a primary effect on the properties and performance of the developed product. These properties are divided into three classes: (1) mechanical properties as strength, toughness, hardness, and ductility; (2) physical properties as thermal and electrical conductivity; and (3) chemical properties as corrosion resistance.

One of the main tasks of modern metallurgy is a joining process that involves the assembling of two or more pieces into one. Joining may be conducted by welding, brazing or soldering techniques. Recent scientific works in this field focus on understanding the physical processes, structural evolution, and the correlation between the created structure and final properties of the metal or alloy. In order to solve complex tasks, knowledge of the interdisciplinary basics in chemistry, physics, mathematics, and engineering is required. In addition to those, the advanced scientific topic of nanoscience is also involved in recent works in order to create material with the highest properties as possible.

An additional task of modern metallurgy is surface engineering, mostly applied in order to improve corrosion and wear resistance. Recent advances in the research of functional coatings and surface engineering focus on the environmentally friendly techniques of fabrication associated with performance improvement and cost-effectiveness. A wide variety of advanced properties may be achieved using different coating technologies, such as bio-inert surfaces, antireflective or antifriction layers, and corrosion resistive coatings, among others.

The current Special Issue contains 21 scientific works that cover recent developments and investigations related to the welding, joining, and coating of metallic materials. These works cover state-of-the-art issues in welding processes, such as resistance spot welding, laser welding, and friction stir welding. The published works are mostly focused on the microstructural study of metallurgy as well as the properties and performance investigation of the created joints. Furthermore, several works on the modeling and calculation of the joints are also presented in this Special Issue.

Microstructural studies and investigations on the properties of different types of steel using welding processes are reported [1,2,3]. Zhang et al. investigate the spot welding process of complex parts conducted on NiTi shape memory alloy using a copper interlayer [4]. These joints may be applicable as biomaterials in the medical industry. A complex process of dissimilar welding is presented by Silvayeh et al. [5], who show numerical calculations of the intermetallic layer thickness of aluminum to steel welding. Statistical analysis of dissimilar welding of aluminum to copper and investigation of the joint microstructure and properties are illustrated by Yang and Jiang [6]. In addition to welding technology, research work on the transient liquid phase bonding approach is published in this Special Issue. AlHazaa and coauthors report the successful bonding between Ti-6Al-4V and Mg AZ31 alloys using a zinc interlayer [7].

As mentioned above, coating technology is also covered by this Special Issue. One of the most promising coating methods in recent years is plasma electrolytic oxidation (PEO) or the micro arc oxidation (MAO) approach, as it is also known. A novel approach of MAO treatment is reported by Sobolev and coauthors who demonstrate oxidation of Ti-based alloy in molten salt for possible biomedical applications [8]. Additionally, other authors also illustrate the influence of MAO parameters on the formation of the oxide coating on Ti-6Al-4V alloy [9]. Other coating approaches are presented by Ali et al. who show stainless steel thin film formation on copper substrates by the physical vapor deposition (PVD) method [10]. Meanwhile, Zenkin et al. implement the chemical vapor deposition (CVD) process on diamond–iron interactions [11]. Chang and Amrutwar show improvements on tool steel mechanical properties using the plasma nitriding method [12].

Finally, we can point out the metallic materials of greatest interest in modern welding, joining, and coating technology whose studies are presented in the current Special Issue. These materials are titanium and magnesium alloys, various types of steel, intermetallics, and shape memory alloys. All of them are attractive to modern science and engineering due to their ability to achieve a combination of advanced properties that open new horizons of application.
